# Diagnostic Value of Blood Oxygen Saturation and Serum C-Reactive Protein Level in Predicting Lung Sequels in Coronavirus Disease 2019-Infected Patients: A 12-week Cohort Study: Oxygen saturation and CRP and COVID-19 lung sequels

**DOI:** 10.31661/gmj.v12i.2695

**Published:** 2023-02-17

**Authors:** Samaneh Abiri, Mina Mohammadizadeh, Lohrasb Taheri, Seyed Reza Mousavi, Masihallah Shakeri, Elahe Rahmanian, Naser Hatami, Erfan Ghanbarzadeh, Zhila Rahmanian, Navid Kalani

**Affiliations:** ^1^ Research Center for Non-communicable Diseases, Jahrom University of Medical Sciences, Jahrom, Iran; ^2^ Student Research Committee, Jahrom University of Medical Sciences, Jahrom, Iran; ^3^ Department of Neurosurgery, School of Medicine, Chamran Hospital, Namazi Teaching Hospital, Shiraz University of Medical Sciences, Iran; ^4^ Student Research Committee, Guilan University of Medical Sciences, Rasht, Guilan, Iran

**Keywords:** Lung, Respiratory, COVID-19, Oxygen

## Abstract

**Background:**

Evidence of Coronavirus disease 2019 (COVID-19) respiratory sequels is restricted and predisposing factors are not well studied more than two years passing pandemic. This study followed COVID-19 patients 12 weeks after discharge from hospital for respiratory sequels.

**Materials and Methods:**

This was a prospective study on discharged COVID-19 patients in 2021, in Jahrom, Iran. Exposure was COVID-19 clinical features at hospitalization, including symptoms and physical examination and laboratory findings, and primary endpoint was 12-week lung sequel, being evaluated by a chest CT scan. Demographics and previous medical history were considered covariates. SPO2 and CRP 6-week changes were followed as an early tool for prediction of 12-week lung sequel.

**Results:**

Totally, 383 participants (17 had sequels) with mean age of 57.43±18.03 years old (50.13% male) completed 12-week study follow-ups. Ninety-one (23.8%) subjects had an ICU admission history. SPO2% in 6th week was statistically significantly associated with a higher rate of 12-week sequelae (P0.001). Also, patients having CT scan scores between 40% to 50% (P=0.012) and higher than 50% (P=0.040) had higher chance of experiencing lung sequelae than patients with CT scan score of below 40%, as well as having ICU admission history and lower SPO2% at 6th week of discharge. There was a statistically significant increasing trend of SPO2% (P0.001) and a statistically significant decreasing trend of CRP levels (P0.001), overall. SPO2% increase after 6 weeks was lower in participants with lung sequels than fully improved ones (P=0.002) and as well as total 12-week change in SPO2% (P=0.001). CRP changes in none of evaluated periods were different among study groups (P0.05).

**Conclusion:**

Our results were in favor of closely following SPO2 levels after patient discharge, while CRP assessment seems not helpful based on our results.

## Introduction

Survivors of the previous coronaviruses of severe acute respiratory syndrome coronavirus (SARS-CoV) and Middle East respiratory syndrome-related coronavirus (MERS-CoV) are reported to experience persistent physiological defects and abnormal radiology findings associated with pulmonary fibrosis[1][2]. Long radiological sequalaes of COVID-19 are also investigated in research [3].

Months after passing the disease course in hospitals, survivors might experience unrestored radiological findings as well as mosaic hypoattenuation [3], ground-glass opacities, Interlobular septal thickening, and reticulation [4].

Longitudinal studies of COVID-19 sequalaes have focused on different physical and functional consequences; while these studies are restricted to not having the baseline data of more advanced tests in follow-ups like pulmonary function tests and functional exercise capacity [5] and small number of participants.

Based on a meta-analysis performed by Huntley et al. suggested abnormal chest CT scan findings in both severe and mild cases [6].

Risk factors of pulmonary decreased function and lung radiological pathology in the largest cohort of COVID-19 survivors were stratified based on the need for oxygen supplementation during the hospitalization, reflecting the severity of the disease [5].

Other potential risk factors of having remained or progressive lung damage is less evaluated and there is no available hallmark or biomarker predicting lung sequalaes .

Salem et al.’s three month cohort for follow up of some biomarkers as well as ESR, platlet count, and D-dimer, was unable to find any assosiation with lung sequales [7]. In this study, we aimed to evaluate the baseline clinical factors of COVID-19 hospitalization course with 12 weeks lung squeal.

## Materials and Methods

This was a prospective cohort study performed on COVID-19 patients who were hospitalized in wards of the Peymanieh Hospital in Jahrom, South of Iran, in 2021. The protocol of this work was authorized with code of "IR.JUMS.REC.1400.036", by the Ethics in Research Committee of the Jahrom University of Medical Sciences. All participants signed the informed consent form.

### Study Population

Sampling was conducted based on the simple-available method from patients being admitted to floor wards. Based on a study with 3.13% radiological lung sequel [8], with an alpha of 0.05 and power of 80%, anticipating 6% lung sequel in our samples, 357 participants were needed as sample size, based on the formula for the prospective study [9].

Inclusion criteria were being hospitalized for COVID-19 and getting discharged after recovery. Also satisfaction for attendance in recalls at six and 12 weeks after discharge in the recruitment center was required for recruitment. COVID-19 was confirmed by nasal swap using polymerase chain reaction (PCR) test.

As we have used high-resolution computed tomography (HRCT) scan at 12 weeks of follow-up, only patients with baseline CT scan records were recruited. Exclusion criteria were death and not attending follow-ups. Patients who had a new respiratory disease, infectious disease, or acute chest syndrome during the 12 weeks of study were also excluded.

To exclude long COVID-19 or reinfection, patients were followed by a nasal swab PCR test performed at 6th and 12th week of discharge, and positive cases were excluded.

We recruited 458 patients. Finally, 383 subjects completed both 6- and 12-week follow-ups with 16.37% loss to follow-up rate.

### Study Outcomes

Exposure was COVID-19 clinical features at hospitalization. These features included symptomatology, physical examinations (PHEs), laboratory data, past medical history (PMHx), and demographic data.

Symptoms were recorded based on the patient reported signs. PHEs were performed by a general practitioner and blood oxygen saturation (SPO2%) was measured at admission time. It was measured by the same Pulse Oximeter device for all patients (BPL Medical Technologies, India). Heart echocardiography was conducted if indicated by a cardiologist and cardiac ejection fraction rate was recorded. Laboratory data on CRP levels were recorded in first blood samples taken at admission. A baseline HRCT was conducted if indicated by the general practitioner’s request. A semi-quantitative CT severity scoring [10] was applied based on the radiologist’s report.

### SPO2 Measurement

Patients were recommended to give rest their bodies for at least 10 minutes before measuring their blood oxygen level. Then, they had to sit up straight, relax, and keep their hands close to the level of heart. The Pulse Oximeter was placed on the tip of index finger, directly on the skin on the index finger of the right hand above the nails and patient had to not move at all while measuring. Three such measurements were performed and highest one was recorded.

### Outcome

12-week lung sequalae was the primary outcome of this study. A chest CT scan was conducted 12 weeks after discharge and reported by the radiologist for any lung abnormality. CRP levels were evaluated at 6 week follow-up and at 12th week of discharge along with the SPO2% measurement.

### Statistical Analysis

Description of study variables was expressed by frequency (relative frequency%) for dichotomous variables and mean±SD for continuous ones. We stratified data based on the quartiles of the CT scan score (Q1: 25th quantile. Q3: 75th quantile) and having or not having lung sequalae in 12th week.

Univariable statistical analyses were performed comparing study variables within the quartiles of the CT scan score or presence of lung sequalae at 12 weeks by independent T-test and Mann-Whitney, ANOVA and Kruskal-Wallis for continuous variables and Chi-square or fisher exact test for binary data.

Repeated measures analysis was conducted to compare data of time-varying variables in 3-time endpoints of T0, or initial referral to hospital, T6 at 6 weeks after discharge from the hospital, and T12 at 12 weeks after discharge.

Multivariate analyses were conducted by assuming lung sequelae at 12th week as the primary outcome using the Cox regression. Variables that had a P value of around 0.2 in univariable comparison of study groups (based on the lung sequalae) were included in multivariate analyses.

A Forest plot of Cox regression was used to visualize the hazard ratio (HR) for each comparison. Changes in SPO2 and CRP were calculated and defined as new variables for different periods and were compared by independent T-test between the groups. All statistical analyses were performed by SPSS version 21. Data were visualized using GraphPad Prism version 8.4.

## Results

In this study, 383 participants were included with mean age of 57.43±18.03 years old. There were 192(50.13%) male subjects. 39.16% of participants had previous cardiac diseases as the most prevalent PMHx; while 35.51% were known as previously healthy.

Dyspnea and cough were most common symptoms (Table- 1). Univariable analysis revealed that all ICU admissions were counted in patients with CT scan score in 4th quartile, showing a statistically significant difference (P<0.001).

Also, patients having 4th quartile CT scan score had the least SPO2% at arrival, compared to other quartiles (P<0.05); while having the highest CRP levels compared to lower quartiles of CT scan score (P<0.05).

There were 366 subjects with no remaining problems in lung and 17 subjects were experiencing lung sequels. None of the compared variables were different in comparison of the patients experiencing lung sequalae versus completely improved ones, except the ICU admission rate that was higher in patients with 12-week sequalae compared to subjects with no lung sequel (52.94% versus 22.4%; P=0.0007), as shown in Table-1.

There was a statistically significant increasing trend of SPO2% (P<0.001) overall and a statistically significant decreasing trend of CRP levels (P<0.001). The type of sequalaes was Reticular in 4 subjects, Septal in 4, Cystic in 6, and fibrosis in 3 subjects.

**Figure-1:**
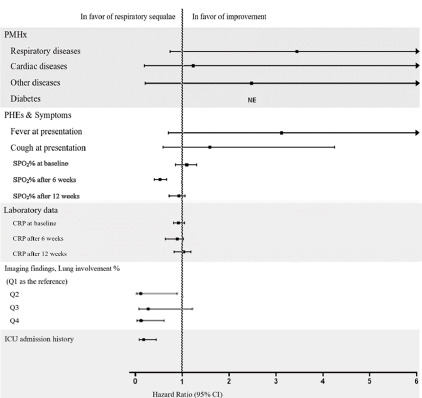
Figure-1. Forest plot of Cox regression for 12th week lung sequelae.
**NE:** not estimated; **Q:** quartile; **PMHx:** past medical history; **PHE**: physical examination.
The plot was visualized by GraphPad Prism 8.

Multivariable analysis considering the 12-week sequalae as the outcome was performed based on the Cox regression. Figure-1 is showing the HR of each variable for 12-week sequalae. Lower SPO2% in 6th week was statistically significantly associated with a higher rate of 12-week sequalae (HR=0.753 [CI95%, 0.684 to 0.828], P<0.001) versus the baseline and 12th week SPO2% (P>0.05). CRP levels in none of the evaluated timelines were predictive of lung sequels (P>0.05). Also, patients having CT scan scores in quartile 2 (HR=0.110 [CI95%, 0.020 to 0.618], P=0.012) and 4 (HR= 0.0.102 [CI95%, 0.11 to 0.902], P=0.040) had a higher chance of experiencing lung sequalae than quartile 1, as shown in Figure-1.

Having ICU admission history was associated with an increased chance of experiencing lung sequalae (HR=0.158 [CI95%, 0.429 to 0.058], P<0.001).

**Figure-2:**
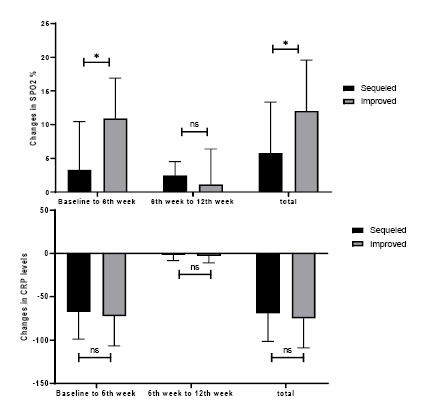
Figure-2. Changes in SPO2% and CRP levels in different timelines of (i) baseline to 6th week; (ii) 6th to 12th week; (iii) total, baseline to 12th week; **ns:** not significant; *: statistical difference.

Based on Figure-2, the amount of SPO2% increase after 6 weeks was lower in participants with lung sequels than fully improved ones (P=0.002) and as well as the total 12-week change in the SPO2% (P=0.001); while between the 6th and 12th week after disease, SPO2% does not change differently among study groups (P=0.783). CRP changes in none of evaluated periods were different among study groups (P>0.05).

## Discussion

Our study was a relatively large sampled-sized cohort of COVID-19 survivors for a Medium-term follow-up period. We found critical COVID-19 patients to be at risk of higher chance of remaining lung radiologic pathology than mild and moderate patients after about 3 months; while none of clinical factors related to pre-existing medical conditions or manifestations at arrival for hospitalization were related. This finding is logically confirmed by many research papers [7].

But the most important finding of this study is the incidence of lung sequalaes in non-ICU admitted patients or mild and moderate COVID-19 patients among 17 patients with lung sequalaes in our study, 8 had not experienced a complicated course of COVID-19. Even patients having baseline lung involvement of lower 40% were also at risk of experiencing lung sequalaes .

This might bring a clinical challenge in predicting and preventing lung sequalaes in COVID-19 patients.

Available tools being used for long-term respiratory follow-up of COVID-19 patients are pulmonary function tests, chest CT scans, and biomarkers [11]; but none are evaluated as monitoring tools. To this aim, after validating adjusted time-varying potential predictors of lung squeal, we evaluated the amount of change in predictors to forecast the incidence of parenchymal lung injury.

The clinical characteristics of patients evaluated in this study are similar to previous reports of COVID-19 patients in Jahrom city [12] and our most evaluated patients were selected within the infected population of city during the 5th wave of disease in the city [13].

In this way, we found that SPO2 changes that are statistically different among the subjects with different severity of lung involvement can be utilized to predict lung sequalaes. SPO2 is also being used as a non-invasive prognostic marker for critically ill patients of COVID-19 [14].

In our study, it was revealed that the amount of improvement of the SPO2 at 6 weeks after discharge can be lower in participants with lung sequels.

Other reports have also suggested that oxygen saturation level can be utilized for follow-up of both severe and mild/moderate COVID-19 cases along with a walk meter test [15]; while as we had not any baseline physical evaluation of the walk meter, we did not include these factors.

We also found that age, need for ICU admission, and symptoms of dyspnea at arrival could affect the SPO2 trend of change during the time. But, the overall decrease of saturation of oxygen after 6 weeks of discharge compared with the baseline admission oxygen saturation is an indication for further investigations, based on our results.

Our study showed a statistically significant decrease in CRP levels during the 12 weeks in almost all patients; while CRP levels were not statistically different between the patients with a long-term respiratory squeal and others, research suggests that its raise is associated with the severity of COVID-19 during the hospitalization [16] and in contrast to our study, some report persistent high CRP levels after 6 weeks of discharge [17].

Another study suggests that physicians should not be awaiting early normalization of laboratory and clinical findings of COVID-19 soon after discharge [18], but no definitive change range is proposed for none of the factors in literature.

As COVID-19 pandemic is getting less intense with the help of broad vaccinations worldwide, cases have tended to manifest with less severity and less need for hospitalization [19][20], management and follow-up of outpatient COVID-19 have earned more respect in 2022, and health systems are trying to get changed to pre-COVID-19 era [21] for utilization of the medical resources as well as the application of the HRCTs for non-COVID patients.

So having available, low-cost, and accurate tools for monitoring COVID-19 outpatients to prevent lung damage is important and we propose following the O2 saturation before conducting early CT scans.

### Strengths and Limitations of Study

This study, having a good sample size, was restricted to some methodological and resource shortage issues. We were just able to radiologically follow the respiratory function of the participants and no pulmonary function tests were available. Our data might have been affected by the effect of the independent radiologists reviewing the HRCT results. Radiologists were not blinded to the primary CT scan record of the patients which might be a source of bias.

## Conclusion

The prevalence of lung sequalaes in patients who were not admitted to the intensive care unit is one of the study’s most significant findings.

Even patients whose baseline lung involvement is less than 40% are susceptible to lung sequalaes.The clinical problem of anticipating and preventing lung sequalaes in COVID-19 patients may result from this. We discovered that it is possible to anticipate lung sequalaes using SPO2 fluctuations that are statistically distinct among participants with varying degrees of lung involvement.

In almost all patients in our study, CRP levels fell statistically significantly throughout the course of the 12 weeks; however, there was no statistically significant difference in CRP levels between individuals with long-term respiratory sequalaes and the other patients.

## Conflict of Interest

The authors declare that there are no conflicts of interest.
